# The association between CCND1 G870A polymorphism and colorectal cancer risk: A meta-analysis

**DOI:** 10.1097/MD.0000000000008269

**Published:** 2017-10-20

**Authors:** Mei Xie, Fen Zhao, Xiaoling Zou, Shuai Jin, Shaoquan Xiong

**Affiliations:** a Department of Oncology, Chengdu University of Traditional Chinese Medicine Affiliated Hospital; b Department of Oncology, Chengdu First People's Hospital, Chengdu, Sichuan, China.

**Keywords:** colorectal cancer, cyclinD1 G870A, meta-analysis, polymorphism

## Abstract

**Background:**

CyclinD1 (CCND1) is a key cell cycle regulatory protein. A large number of epidemiological studies have assessed the potential correlation between the CCND1 G870A polymorphism and the risk of colorectal cancer (CRC), but their findings have been inconsistent. To obtain a more precise understanding of the association between the G870A polymorphism in the CCND1 gene and the CRC risk, we conducted a more comprehensive meta-analysis.

**Methodology:**

We searched PubMed, Ovid, Springer, Weipu, China National Knowledge Infrastructure (CNKI), and Wanfang databases, covering all publications (the last search was updated on January 10, 2017). The pooled odds ratios (ORs) with 95% confidence intervals (CIs) were derived from a fixed effect or random effect model. Statistical analyses were performed using Review Manager 5.3 and STATA 10.0 software.

**Results:**

A total of 7276 CRC patients and 9667 controls from 27 publications were included in this meta-analysis. We found that compared with GG homozygote genetic model, AA, AG, AA + AG genetic models of the CCND1 G870A polymorphism were significantly associated with overall CRC risk (AA homozygote genetic model: OR = 1.28, 95% CI = 1.10–1.49; AG heterozygote genetic model: OR = 1.15, 95% CI = 1.06–1.25; AA homozygote + AG heterozygote genetic model: OR = 1.19, 95% CI = 1.07–1.33). Subgroup analyses by ethnicity and cancer location showed that A carriers were consistently associated with a significantly increased risk of CRC in all subsets of participants (Asian and Caucasian; colon cancer and rectal cancer). When stratified by study design, we found a significant association in hospital-based studies (HB), but no significant associations were found in either population-based studies (PB) or family-based studies (FB). According to subgroup analysis by cancer type, the risk of sporadic colorectal cancer (sCRC) and hereditary nonpolyposis colorectal cancer (HNPCC) were not correlated with the CCND1 G870A polymorphism, except AG (AG vs GG: OR = 1.30, 95% CI = 1.11–1.53).

**Conclusions:**

This meta-analysis suggests that the CCND1 G870A polymorphism is associated with an increased risk of CRC, especially that A carriers may be a major risk factor for CRC.

## 1 Introduction

Colorectal cancer (CRC) is a common malignant tumor of the digestive tract, which has become a serious threat to human health. Globally, there were an estimated 1.36 million new cases of CRC and 694,000 deaths in 2012.^[1]^ The global number of CRC cases is expected to increase by 60% to more than 2.2 million new cases and approximately 1.1 million deaths annually by 2030.^[2]^ Although effective therapeutic strategies have been developed over the past decades, the 5-year overall survival of CRC still remains unsatisfactory because of the presence of poor prognostic factors such as vascular and neural invasion, a low lymphocyte-to-monocyte ratio (LMR), and tumor stage III/IV.^[1]^ The economic burden of CRC is substantial. Particularly, the long-term cost of CRC causes huge social burden.^[3]^ Welch and Robertson^[4]^ provided evidence that population aged 50 years or older had a steady decline in colorectal cancer. However, according to the same data source, the incidence had steadily increased among people younger than 50 years.^[5]^ Therefore, it is extremely important to find the risk factors that can lead to CRC except for advanced age. Factors including environment, life style, diet habit, and others all contribute to the development of CRC.^[6,7]^ Several environmental factors have attributed to the incidence of CRC is more than 85%,^[8]^ especially smoking, drinking, meat consumption, exposure to aryl amines, and heterocyclic amines.^[9]^ Approximately 20% of CRC patients have a family history of cancer, indicating that genetic factors may play a role in CRC susceptibility.^[10–12]^ And the discovery is also evidence supporting that the disease has polygenic and multiple-factorial bases.^[13]^

Cancer is a genetic and cell-cycle disease, its occurrence and development involve a multistep and polygenic process.^[14]^ CyclinD1 (CCND1) is a key cell cycle regulatory protein, and its expression and cellular localization is often transformed in human tumor cells. CCND1 is the gate keeping protein that charges regulating the transition through the restriction point in the G1 phase to S phase of the cell cycle. So the mechanisms of CCND1 gene amplification, posttranscriptional or posttranslational modifications, rearrangements, and variant polymorphisms can lead to abnormal protein levels and result in risk of cancer.^[15–18]^ The common guanine-to-adenine polymorphism at nucleotide position 870 of the CCND1 gene is known to modulate the frequency of alternate splicing and presumably reduce transcript levels.^[19]^

It has been demonstrated in recent studies that high levels of CCND1 protein expression are related to poorer outcomes in patients with CRC.^[20,21]^ And there were many case-control studies that have evaluated the potential impact of CCND1 (G870A) gene polymorphism on the risk of CRC,^[22,23]^ and meta-analyses have also been performed to investigate the association between the CCND1 G870A polymorphism and the CRC risk.^[24–27]^ Notwithstanding, their findings remain inconclusive and controversial. Therefore, we conducted this current meta-analysis to provide more compelling evidence for the relationship between the CRC risk and the CCND1 G870A polymorphism.

## 2 Materials and methods

### 2.1 Search strategy

We searched 6 online databases including PubMeb, Ovid, Springer, Weipu, China National Knowledge Infrastructure (CNKI), and Wanfang databases (the last search was updated on January 10, 2017). We used a search strategy of Me-SH terms and keywords: “Colorectal Neoplasms or Colon Neoplasms or Rectal Neoplasms or Colorectal cancer or Colon cancer or Rectal cancer” and “CyclinD1 or CCND1 or CyclinD1 G870A or CCND1 G870A” and “Polymorphism, genetic or Polymorphism.” The search was restricted to English and Chinese publications.

### 2.2 Eligibility criteria

The inclusion criteria of studies in our meta-analysis were as follows: studies that evaluated the impact of the G870A polymorphism in the CCND1 gene on the risk of CRC; studies that used a case-control design; studies with sufficient data (genotype distributions for both patients and controls); and genotype distributions of the control population must be consistent with Hardy–Weinberg equilibrium (HWE). The major exclusion criteria were: no control group was included; genotype frequencies or number were not reported; or reviews, abstracts, and duplicate studies.

### 2.3 Data extraction

Two reviewers independently and carefully extracted the information from all selected publications. Also they reached a consensus on all the items. If the 2 authors had a debate about the selected studies, a third author would adjudicate disputes. For each selected study the following items of information were extracted and tabulated: first author, year of publication, original country, ethnicity (Caucasian, Asian, or Mixed), study design (population-or hospital-or family-based study, PB, HB, or FB), type of CRC (hereditary nonpolyposis colorectal cancer (HNPCC), sporadic colorectal cancer (sCRC), or mixed) and location of CRC (colon cancer, rectal cancer, or mixed), genotyping methods (polymerase chain reaction (PCR) single-stranded conformation polymorphism (PCR-SSCP), PCR restriction fragment length polymorphism (PCR-RFLP), high-performance liquid chromatography (HPLC), TaqMan PCR, or Multiplex PCR), as well as the number of patients and controls.

### 2.4 Statistical analysis

For each study, the genotype distribution was tested in controls, which was based on HWE using an Internet-based program. The association between the CCND1 G870A polymorphism and the risk of CRC was evaluated by crude ORs with 95% CI. We assessed the CRC risk of individuals with genotype AA versus GG, AG versus GG, AA and AG versus GG, A versus G, respectively. We performed all statistical analyses of the meta-analysis by using Review Manager Version 5.3. A *P* value of less than.05 was considered statistically significant. Heterogeneity was checked by a *χ*^2^-based Q statistic among the included studies. When the consequence was *P* > .10 for the Q-test indicating a lack of heterogeneity among studies, the fixed–effects model was used to calculate the pooled OR; otherwise, the pooled OR was calculated by the random effect model. We performed stratified analyses by ethnicity, location of CRC, study design, and type of CRC, so as to evaluate their specific effects on the risk of CRC. The subgroup analysis by ethnicity was classified as Caucasian, Asian, or Mixed (when the participants were difficult to be divided into Asian or Caucasian, the study was termed “Mixed”). The different classification according to location of CRC was: colon cancer, rectal cancer, or mixed (the specific location was not mentioned). The study design was described as HB, PB, or FB. To evaluate the effect of CRC type, the participants were stratified into sCRC, HNPCC, or mixed (included both sCRC and HNPCC, or the specific type was not mentioned).

Moreover, sensitivity analysis was performed to assess the stability of the results, by orderly excluding individual studies. Publication bias was analyzed by Begg funnel plot and Egger test.^[28,29]^ All statistical analyses were performed using Review Manager 5.3 and STATA 10.0 software.

## 3 Results

### 3.1 Study inclusion and characteristics

**Figure 1:**
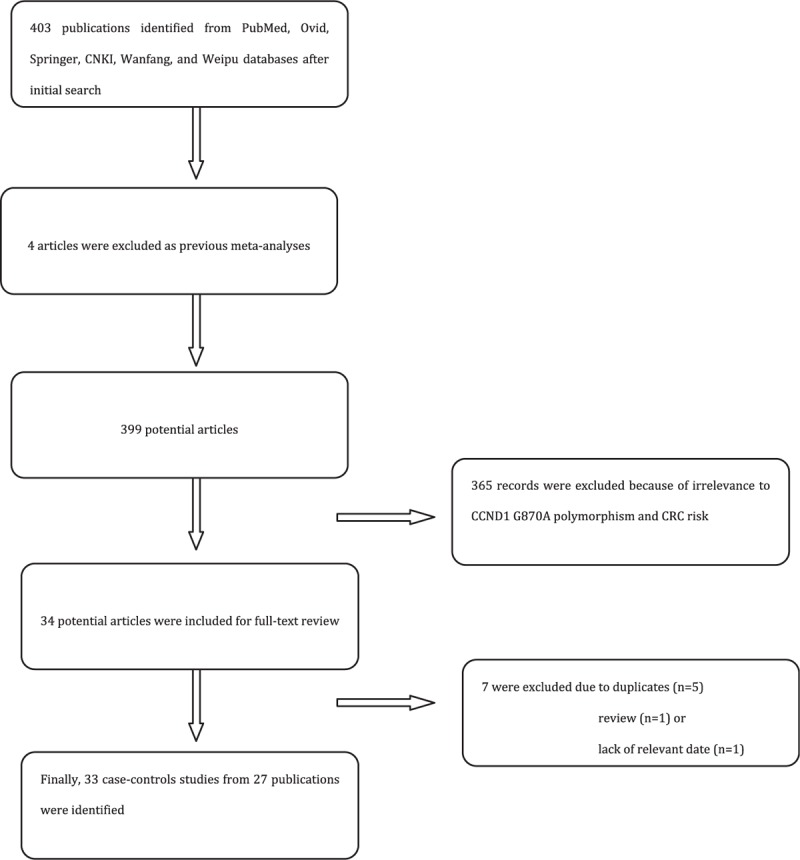
Flow diagram of included/excluded studies.

As shown in Fig. 1, we searched PubMed, Ovid, Springer, CNKI, Wanfang, and Weipu database. Initially, a total of 403 results were identified on CCND1 and CRC. Then 4 articles were excluded as previous meta-analyses. After reading the titles and abstracts, 365 were excluded because they were irrelevant to CCND1 G870A polymorphism and 34 potential articles were included for full-text review. After reading the full texts, 7 articles were excluded to duplicates (n = 5), review (n = 1), or lack of the relevant date (n = 1). Finally, a total of 33 case-control studies from 27 publications^[14,22,23,30–53]^ which met our inclusion criteria, including 7276 cases and 9667controls. The main characteristics of each study identified are listed in Table 1. Briefly, 12 case-control studies were performed in Asians,^[22,23,36–38,40,41,44,45,50,52,53]^ 12 in Caucasians,^[14,31–36,39,42,46,47,51]^ 5 in Mixed.^[30,36,43,48,49]^ As for study design, there were 2 FB,^[30,31]^ 14 PB,^[14,23,32,33,36,37,39,41–45,48,50,53]^ and 11 HB.^[22,34,35,38,40,43,46,47,49,51,52]^ Eleven studies described the specific location of CRC.^[14,22,23,36,38,40,43,48,50,52,53]^ Genotype and allele distributions for each case-control study are shown in Table 2.

**Table 1:**
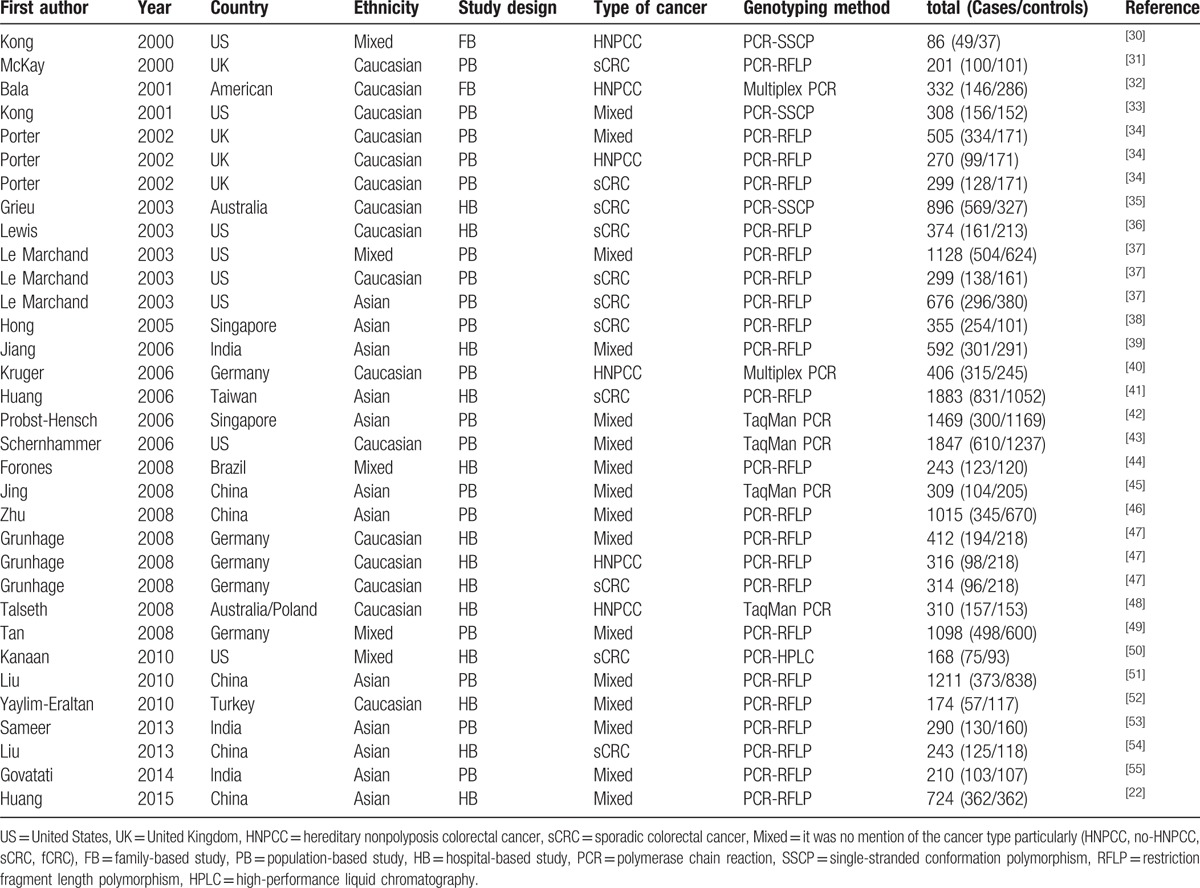
Characteristics of the studies included in meta-analysis.

**Table 2:**
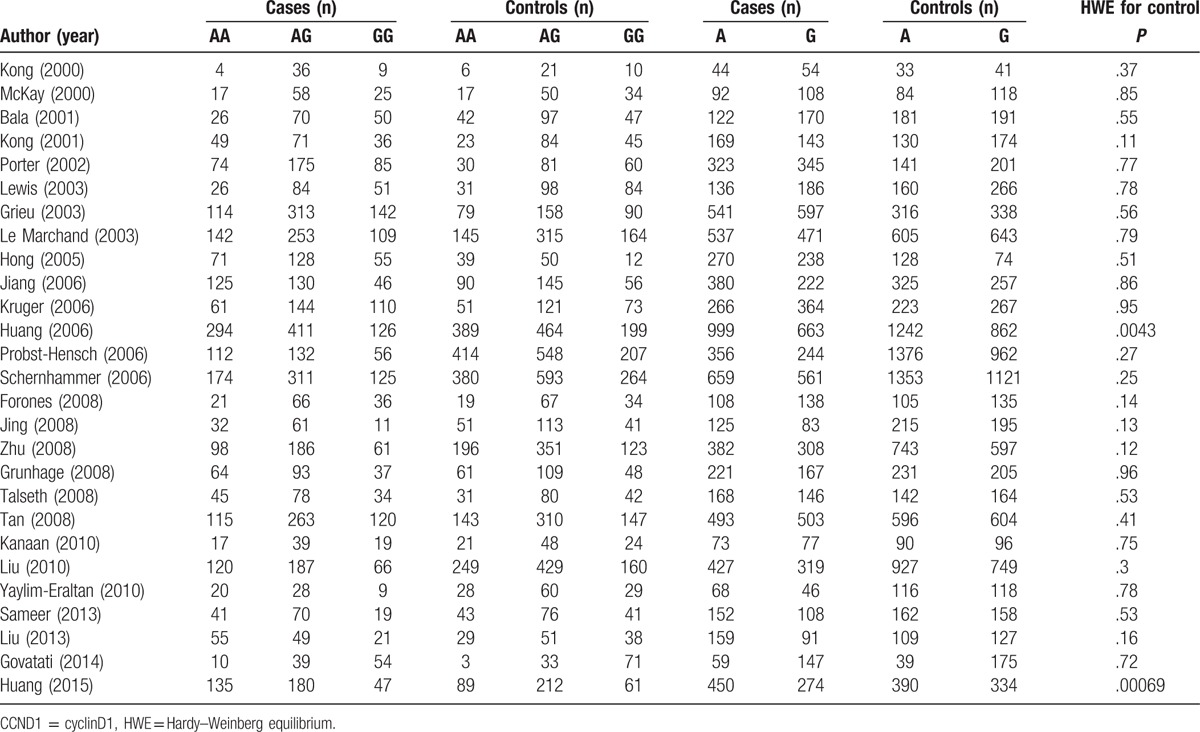
Distribution of CCND1 G870A genotype and Allele among colorectal cancers and controls.

### 3.2 Main meta-analysis results

**Figure 2:**
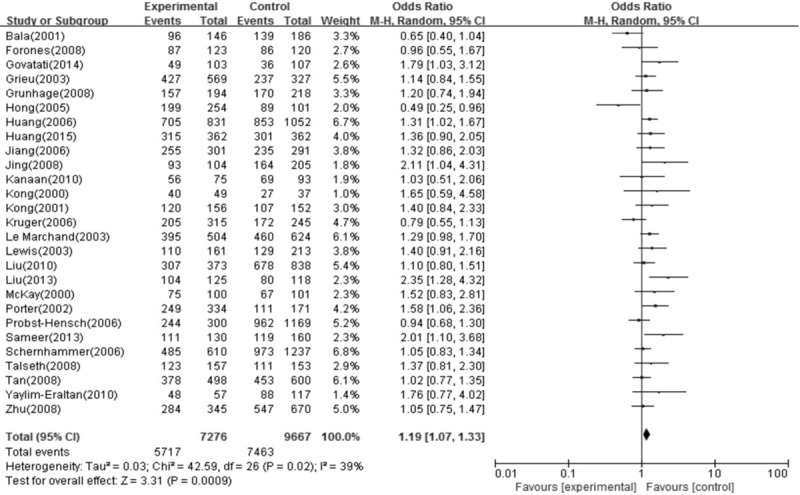
Meta-analysis of association between CCND1 G870A polymorphism and colorectal cancer (AA and AG versus GG) when all the subjects in the 27 studies were included (Events: AA + AG; Total: AA + AG + GG). CCND1 = cyclinD1.

The heterogeneity between AA + AG versus GG was assessed for the 33 studies (*P* = .02) and the *χ*^2^ value was 42.59 with 26 degrees of freedom (Fig. 2). Therefore, a random-effects model was used for the synthesis of data. The overall OR was 1.19 (95% CI = 1.07–1.33) and the *Z* test value for overall effect was 3.31 (*P* = .0009). The results suggested that the variant A allele carriers had a 19% increased risk of CRC. We also found that compared with GG homozygote, AA homozygote, or AG heterozygote of the CCND1 G870A polymorphism was significantly associated with a higher overall risk for CRC (AA vs GG: OR = 1.28, 95% CI = 1.10–1.49; AG vs GG: OR = 1.15, 95% CI = 1.06–1.25). Summary results of other genetic comparisons are listed in Table 3.

**Table 3:**
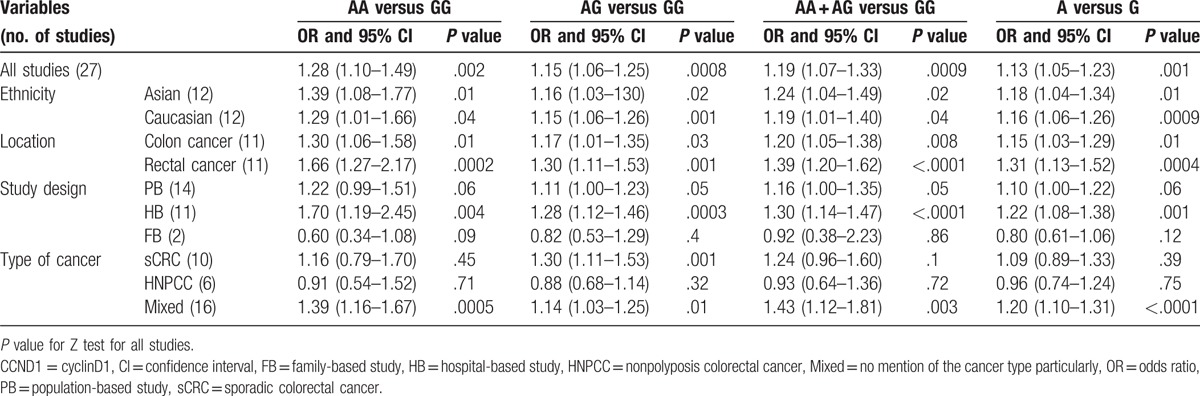
Association between CCND1 G870A polymorphism and colorectal cancer.

### 3.3 Subgroup analyses

**Figure 3:**
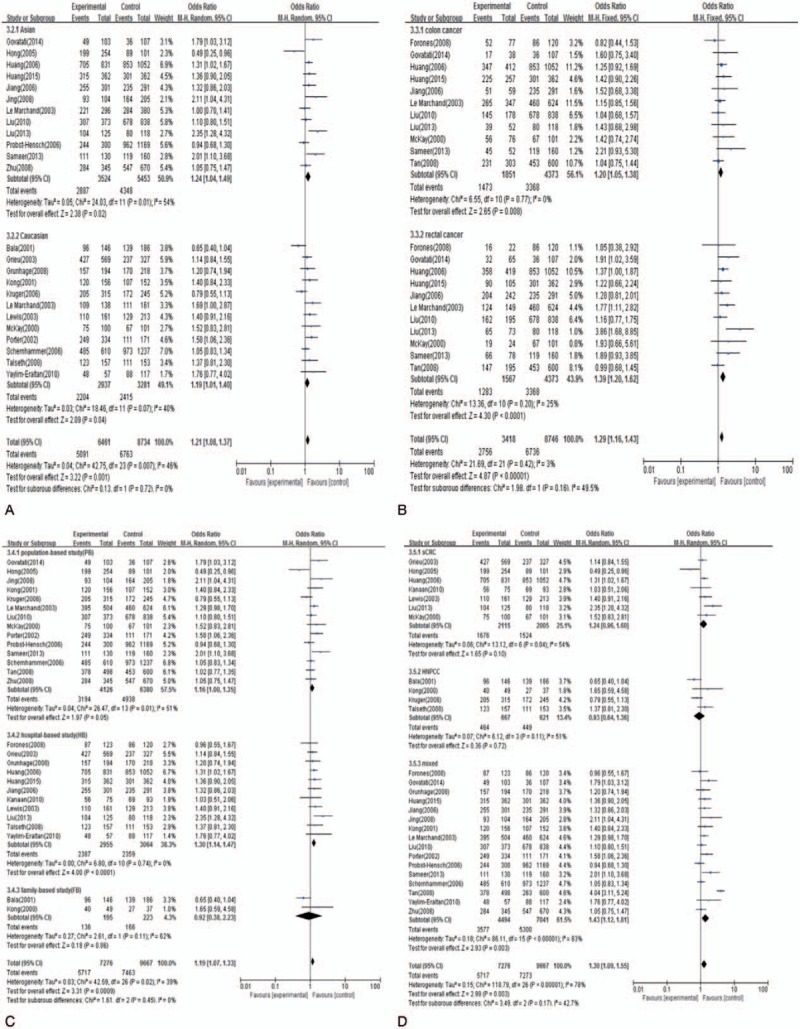
A, Meta-analysis of the association between CCND1 G870A polymorphism and CRC in Ethnicity. B, Meta-analysis of the association between CCND1 G870A polymorphism and CRC in location. C, Meta-analysis of the association between CCND1 G870A polymorphism and CRC in study design. D, Meta-analysis of the association between CCND1 G870A polymorphism and CRC in cancer type. CCND1 = cyclinD1, CRC = colorectal cancer.

We performed subgroup analyses by ethnicity (Asian or Caucasian), location of CRC (colon cancer or rectal cancer), study design (PB, HB, or FB), and type of CRC (sCRC or HNPCC). Using GG genotype as a reference, A carriers were associated with a significantly increased risk of CRC in both Asians (AA + AG vs GG: OR = 1.24, 95% CI = 1.04–1.49) and Caucasians (AA + AG vs GG: OR = 1.19, 95% CI = 1.01–1.40). This indicated that A carriers might be a low-penetrant risk factor for CRC in both Asian and Caucasian populations. When stratified by cancer location, significant associations between A carriers and CRC risk were found in both subsets of patients with colon cancer (AA + AG vs GG: OR = 1.20, 95% CI = 1.05–1.38) and rectal cancer (AA + AG vs GG: OR = 1.39, 95% CI = 1.20–1.62). Subgroup analysis by study design indicated that significant association between the CCND1 G870A polymorphism and the risk of CRC was only observed in HB studies (AA + AG vs GG: OR = 1.30, 95% CI = 1.14–1.47), rather than PB (OR = 1.16, 95% CI = 1.00–1.35) or FB studies (OR = 0.92, 95% CI = 0.38–2.23). According to analysis by cancer type, no significant association was noted between the CCND1 G870A polymorphism and an increased risk of CRC in patients with sCRC (AA + AG vs GG: OR = 1.24, 95% CI = 0.96–1.60) and HNPCC (AA + AG vs GG: OR = 0.93, 95% CI = 0.64–1.36), but a significantly increased CRC risk was found in sCRC patients with genotype AG (AG vs GG: OR = 1.30, 95% CI = 1.11–1.53) (Fig. 3, Table 3).

### 3.4 Sensitivity analysis and publication bias

**Figure 4:**
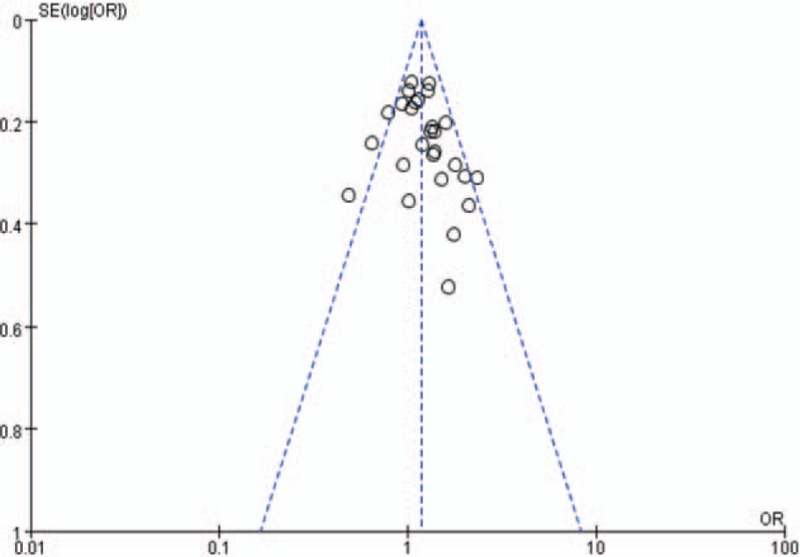
Funnel plot analysis for odds ratios of AA and GG genotype compared with GG genotype in overall studies.

We performed a sensitivity analysis through sequentially excluded individual studies. No individual study affected the overall OR dominantly, statistically similar results were obtained, suggesting the stability of this meta-analysis (data not shown). We used the Begg funnel plot and the Egger test. The shape of the funnel plots of the 27 publications appeared symmetrical for the AA + AG versus GG model (Fig. 4), indicating no evidence of significant publication bias in this meta-analysis. And the Egger test results also supported that there was no evidence of publication bias (*P* > .05).

## 4 Discussion

CRC is the third most common cancer in both men and women across the world.^[1]^ To date, the pathogenesis of CRC has not yet been fully clarified. Several risk factors such as age, environment, high-fat diet, and heredity have been recognized. In recent years, numerous studies have revealed a direct relationship between the CCND1 gene and tumors, including lymphoma,^[54,55]^ breast cancer,^[56,57]^ lung cancer,^[58,59]^ bladder cancer,^[60,61]^ and colorectal cancer.^[62,63]^ It has been demonstrated that the over-expression of CCND1 may collaboratively participate in cancer carcinogenesis.

As we know, CCND1 has been considered to be a cancer gene which could regulate progression from the G1 phase of the cell cycle to the S phase. Cells with the mutant allele accumulate mutations as a result of defective mismatch repair and bypass the G1-S checkpoint of the cell cycle more easily than in cells not carrying the polymorphism.^[30]^ Variant polymorphisms can result in abnormal protein levels and lead to cancer.^[20]^ The CCND1 over expression has been reported to occur in 72% of colorectal tumors.^[62]^ However, results of case-control studies about this genetic polymorphism were inconsistent. A few meta-analyses^[24–27]^ were also designed to confirm the influence of CCND1 G870A polymorphism on CRC susceptibility. These analyses found that A carriers of the CCND1 G870A polymorphism were significantly associated with an increased risk of CRC. However, their detailed descriptions on ethnicity, cancer location, study design, and family history varied significantly. Zou et al,^[26]^ the most recent study published in 2012 included 23 case-control studies, and concluded that the CCND1 870A allele might be a low-penetrant risk factor for CRC. The result was consistent with the findings reported by Yang et al^[24]^ and Zhang et al^[27]^. But in further stratified analyses by ethnicity and study design, such a correlation was not found in any subsets of participants. This result was contradictory to those from the other 3 meta-analyses,^[24,25,27]^ which observed an increased risk in the subgroups of sCRC and in Caucasians.

This meta-analysis of 33 case-control studies supported that the G870A polymorphism of CCND1 was a risk factor for CRC. A allele carriers had a 1.19-fold elevated risk of CRC. As previous studies reported inconsistent findings in their subgroup analyses, we performed more robust stratified analyses to comprehensively analyze these subset associations. Our findings revealed that the CCND1 G870A polymorphism was associated with an increased risk of CRC in both Asian and Caucasian. Meanwhile, such an association was also observed in subsets of either cancer location (colon cancer and rectal cancer). As for the study design, we found a significant association in HB studies, but not in PB or FB studies, which was consistent with a previous meta-analysis.^[24]^ When stratified by type of CRC, no relationship was identified between the CCND1 G870A polymorphism and the risk of CRC in subsets of patients with either sCRC or HNPCC. We speculated that this might be explained by the differences in case-control conditions, genetic classification method, living environment, genetic background, tumor stage, and/or living habits among the included studies.

It has been shown in prior studies that CCND1 870A allele carriers had been confirmed that may be an increase the risk of developing esophageal cancer and hepatocellular carcinoma.^[64,65]^ In this study, we come to a similar conclusion that the CCND1 G870A polymorphism is a potential factor of CRC. However, a few meta-analyses^[66,67]^ reported that the CCND1 G870A polymorphism may not be associated with an increased risk factor for cervical cancer and head and neck cancer. Perhaps this is due to similar CCND1 gene expressions in gastrointestinal carcinomas rather than tumors of other systems.

Compared with previous meta-analyses, we found a significant association between the CCND1 G870A polymorphism and the CRC risk in many different subgroups. We inferred that 3 reasons might explain the different results between our study and prior studies. First, a larger number of case-control studies were included in our meta-analysis than previous studies, so our conclusion seemed to be more powerful and reliable. Second, no conspicuous publication bias was detected in our study, which indicated that the entire pooled results might be unbiased. Third, comparisons of all genetic models were performed in our study, suggesting that this polymorphism analysis might be more comprehensive and credible.

We also acknowledge several limitations of this meta-analysis. First, we only selected articles published electronically in 6 databases, so it is possible that some pertinent studies not included in these databases or unpublished studies with negative results may have been missed. Second, as participants in the control groups were selected from healthy persons or patients, there might be a lack of proper matching of controls in the included studies, which is likely to influence the consistency of our results. Third, only small numbers of participants were included in some subgroups such as subsets of FB studies and HNPCC patients. Therefore, these subgroup analyses may not have enough statistical power with the small sample size and the conclusions may be biased.

In conclusion, this meta-analysis demonstrated that the CCND1 G870A polymorphism may be associated with an increased risk of developing CRC. Subgroup analyses by ethnicity, cancer location, and study design revealed significant associations between the CCND1 G870A polymorphism and CRC susceptibility in A carriers (AA or AG or AA + AG), especially among Asian and Caucasian populations, patients with colon cancer or rectal cancer, and in hospital-based studies. This may provide a vital theoretical basis to understand the effect of the CCND1 G870A polymorphism on the pathogenesis of CRC. As the CCND1 G870A may play an important role in predicting the occurrence and progression of CRC. Our findings may provide valuable insights into the development of novel diagnostic approaches, gene-targeted therapies, and prevention strategies to combat against CRC. Considering the above-mentioned limitations, larger-scale and well-designed studies are still required to further validate these findings and investigate an even wider range of associations in the future.
